# Efficacy, Safety and Tolerability of Pyronaridine-artesunate in Asymptomatic Malaria-infected Individuals: a Randomized Controlled Trial

**DOI:** 10.1093/cid/ciab425

**Published:** 2021-05-13

**Authors:** Edgard D Dabira, Sebastian Hachizovu, Bakary Conteh, Alieu Mendy, Haddy Nyang, Bolarinde Lawal, Mamadou Ousmane Ndiath, Joyce M Mulenga, Sydney Mwanza, Isabelle Borghini-Fuhrer, Sarah Arbe-Barnes, Robert Miller, Jangsik Shin, Stephan Duparc, Umberto D’Alessandro, Christine Manyando, Jane Achan

**Affiliations:** 1 Disease Control and Elimination Theme, Medical Research Council Unit, The Gambia at London School of Hygiene & Tropical Medicine, Fajara, The Gambia; 2 Tropical Diseases Research Centre, Ndola, Zambia; 3 Medicines for Malaria Venture (MMV), Geneva, Switzerland; 4 Artemida Pharma Ltd, Stevenage, United Kingdom; 5 Shin Poong Pharmaceutical Co, Ltd, Seoul, Korea

**Keywords:** pyronaridine-artesunate, malaria, asymptomatic, pediatric, randomized controlled clinical trial

## Abstract

**Background:**

Pyronaridine-artesunate (PA) is a registered artemisinin-based combination therapy, potentially useful for mass drug administration campaigns. However, further data are needed to evaluate its efficacy, safety and tolerability as full or incomplete treatment in asymptomatic *Plasmodium falciparum*-infected individuals.

**Methods:**

This phase II, multi-center, open label, randomized clinical trial was conducted in The Gambia and Zambia. Participants with microscopically confirmed asymptomatic *P. falciparum* infection were randomly assigned (1:1:1) to receive a 3-day, 2-day, or 1-day treatment regimen of PA (180:60 mg), dosed according to bodyweight. The primary efficacy outcome was polymerase chain reaction (PCR)-adjusted adequate parasitological response (APR) at day 28 in the per-protocol population.

**Results:**

A total of 303 participants were randomized. Day 28 PCR-adjusted APR was 100% for both the 3-day (98/98) and 2-day regimens (96/96), and 96.8% (89/94) for the 1-day regimen. Efficacy was maintained at 100% until day 63 for the 3-day and 2-day regimens but declined to 94.4% (84/89) with the 1-day regimen. Adverse event frequency was similar between the 3-day (51.5% [52/101]), 2-day (52.5% [52/99]), and 1-day (54.4% [56/103]) regimens; the majority of adverse events were of grade 1 or 2 severity (85% [136/160]). Asymptomatic, transient increases (>3 times the upper limit of normal) in alanine aminotransferase/aspartate aminotransferase were observed for 6/301 (2.0%) participants.

**Conclusions:**

PA had high efficacy and good tolerability in asymptomatic *P. falciparum-*infected individuals, with similar efficacy for the full 3-day and incomplete 2-day regimens. Although good adherence to the 3-day regimen should be encouraged, these results support the further investigation of PA for mass drug administration campaigns.

**Clinical Trials Registration:**

NCT03814616.

## Teaser

This randomized study in The Gambia and Zambia evaluated pyronaridine-artesunate as full or incomplete treatment in individuals with asymptomatic *Plasmodium falciparum* infection. High efficacy and good tolerability suggest that pyronaridine-artesunate could be useful in mass drug administration campaigns in Africa.

In 2015, the World Health Organization (WHO) Global Technical Strategy set ambitious goals for reducing malaria mortality and incidence rates by at least 90% and achieving malaria elimination in at least 35 countries by 2030 [1]. Eleven countries worldwide, 10 of them in sub-Saharan Africa, contribute about 70% of global malaria morbidity and mortality [2]. Even in areas with high coverage of control interventions, malaria transmission persists and has become increasingly heterogeneous [3–5]. Innovative tools and strategies are needed to reduce malaria transmission and promote elimination.

A major challenge for malaria elimination is transmission from asymptomatic malaria-infected individuals carrying low-density infections [6–8]. Interventions targeting the human transmission reservoir, such as mass drug administration (MDA), can reduce malaria prevalence and transmission [9–16]. Effective MDA requires high coverage and good adherence to treatment [17–19], and there is a need for efficacious, well-tolerated, and affordable treatment for this purpose.

Pyronaridine-artesunate (PA) is a fixed-dose artemisinin-based combination therapy (ACT) shown to be highly efficacious and well tolerated for the treatment of uncomplicated falciparum malaria [20–32]. This study is the first to our knowledge to evaluate PA efficacy, safety, and tolerability in individuals with asymptomatic *Plasmodium falciparum* infection. To assess the potential impact of suboptimal adherence on parasitological efficacy, PA was administered at the full therapeutic dose (once daily for 3 days) and as incomplete treatment (once daily for 2 days or 1 day).

## METHODS

### Ethics Statement

The protocol was approved by the Gambian Government/MRC Joint Ethics Committee in The Gambia, the Tropical Diseases Research Centre (TDRC) Ethics Review Committee and the National Health Research Ethics Board in Zambia, and the Ethics Committee of the London School of Hygiene and Tropical Medicine. The study was conducted in accordance with the Declaration of Helsinki, Good Clinical Practice, and applicable national regulations. Written informed consent was obtained from all patients or their parents/guardians if aged <18 years; documented assent was obtained from children aged 12–17 years.

### Study Design and Participants

This phase II, multicenter, open label, randomized clinical trial was conducted in Basse (Upper River Region), Eastern Gambia, and Nchelenge (Luapula Province), Northern Zambia, between 2 October 2018 and 16 May 2019. Trial sites were in areas of moderate-to-high malaria transmission. Potential study participants were identified by systematic prescreening for malaria infection in local communities and schools until the required sample size was reached.

Inclusion criteria were confirmed *P. falciparum* mono-infection with a parasite density between 20 and 50 000/µL, no clinical malaria signs or symptoms for the past 72 hours, age >5 years, body weight >20 kg, and the ability to swallow oral medication. Participants were excluded if they had a hemoglobin level <7 g/dL, evidence of severe malnutrition, known allergy to the study drugs. Complete eligibility criteria are described in Supplementary Methods 1.

### Study Drug

Pyronaridine-artesunate (180/60 mg) fixed-dose combination tablets (Shin Poong, Pharmaceutical, Co, Ltd) were given orally, once daily, according to body weight: 20 to <24 kg, 1 tablet; 24 to <45 kg, 2 tablets; 45 to <65 kg, 3 tablets; and ≥65 kg, 4 tablets. Treatment was administered for 3 days (3-day regimen), 2 days (2-day regimen), or 1 day (1-day regimen). All doses were directly supervised. Vomiting within 30 minutes prompted repeat dosing. Vomiting of the repeat dose resulted in participant withdrawal and rescue treatment as per local recommendations.

### Randomization and Masking

Participants were randomized (1:1:1) to receive the PA 3-day regimen, 2-day regimen, or 1-day regimen according to a computer-generated randomization list provided by the study sponsor. Treatment allocation was in sealed envelopes sequentially numbered with the study participant’s unique code. Participants were allocated in enrolment order to the treatment in the next available envelope. Participants and clinical staff were not masked to treatment regimen; microscopists responsible for reading malaria smears remained blinded to treatment allocation throughout the study.

### Procedures

Pre-screening for malaria infection was done using a standard rapid diagnostic test (RDT; SD Bioline Malaria Ag Pf, Standard Diagnostics Inc.) or hypersensitive (HS)-RDT (Alere Malaria Ag Pf, Standard Diagnostics, Inc.) in Zambia and HS-RDT in The Gambia, with confirmation by microscopy. Eligible participants received their first PA dose on day 0; a blood slide was collected 4–8 hours after the first dose. Participants returned on days 1, 2, 3, 7, 14, 21, 28, 35, 42, and 63, or at any time if they felt unwell. Insecticide-treated bed nets were provided to all participants on day 0. The assessment schedule is shown in Table 1.

**Table 1.** Assessment Schedule

	Study Day/ Visit												
Assessment	BL	D0^a^	D1	D2	D3	D7	D14	D21	D28	D35	D42	D63	EW/UV
Demographics, medical history	●												
Urine pregnancy test	●								●			●	●
Physical examination^b^	●		●	●	●	●	●	●	●	●	●	●	●
Thick/thin blood smears	●	●	●	●	●	●	●	●	●	●	●	●	●
Blood spot (PCR genotyping)^c^	●					● ^d^	● ^d^	● ^d^	● ^d^	● ^d^	● ^d^	● ^d^	●
Hematology/biochemistry	●		●			●			●				●
Adverse events	●	●	●	●	●	●	●	●	●	●	●	●	●
Concomitant medication	●	●	●	●	●	●	●	●	●	●	●	●	●
Study drug administration	●		●	●									

Abbreviations: BL, baseline; EW, early withdrawal; EW, Early Withdrawal; PCR, polymerase chain reaction; UV, unscheduled visit.

^a^4–8 hours.

^b^Physical examination, malaria signs and symptoms, vital signs, and body temperature.

^c^Increases in aspartate aminotransferase, alanine aminotransferase, alkaline phosphatase, total or conjugated bilirubin >3 times the upper limit of normal (×ULN) prompted collection of an additional sample within 24 h and repeated sampling at 48-h intervals until values were ≤2×ULN.

^d^Assessment was only done in the event of recurrent infection.

Giemsa-stained thick and thin blood smears for parasite identification and quantification were examined independently by 2 microscopists using standard methods [33]. Any discordant blood smears or those with >30% variance in parasite density were reviewed independently by a third microscopist, with external quality control on approximately 4% of slides. To distinguish between recrudescence and re-infection, blood spots were obtained for *P. falciparum* polymerase chain reaction (PCR) genotyping. Recrudescence was defined as at least 1 matching allelic band in all markers (*P. falciparum* genes *msp 1*, *msp 2*, and *glurp*) between samples from baseline and recurrence [34].

Demographic characteristics were recorded, and a medical history taken at screening. Physical examination, vital signs, malaria signs and symptoms, and adverse events were assessed throughout the study and categorized using the Medical Dictionary for Regulatory Activities (version 22.1). Blood samples were collected for hematology and clinical chemistry.

### Outcomes

The primary efficacy outcome was day 28 PCR-adjusted adequate parasitological response (APR), defined as a microscopically negative slide at day 28, irrespective of axillary temperature, in participants without previous treatment failure. Secondary efficacy endpoints were: i) PCR-adjusted APR at days 7, 14, 21, 35, 42, and 63; ii) PCR-unadjusted APR at days 7, 14, 21, 28, 35, 42, and 63; iii) recurrence, reinfection and recrudescence incidence rate until day 63; iv) the proportion of participants parasite-free by microscopy between 4 and 8 hours post first PA dose and by day 1, 2, and 3 post-first dose; and v) gametocyte carriage up to day 14, by microscopy.

Safety outcomes were adverse event frequency, and abnormal vital signs, hematological parameters, or clinical chemistry values. Serious adverse events were defined as death, life-threatening, requiring hospitalization or prolongation of hospitalization, congenital abnormalities, or birth defects, persistent or significant disability or incapacity, or Hy’s law (alanine aminotransferase [ALT] or aspartate aminotransferase [AST] >3 times the upper limit of normal [×ULN] plus a serum total bilirubin >2×ULN [>35% direct bilirubin], in the absence of alkaline phosphatase ≥2×ULN or biliary injury).

### Sample Size

The 3-day regimen was assumed to have similar efficacy against *P. falciparum* in asymptomatic carriers as in patients with uncomplicated malaria, that is, ≥97% at day 28 [20, 31, 32]. With a sample size of 90 participants, assuming an efficacy of 97.8% for the 3-day regimen, the lower limit of the 1-sided Clopper–Pearson 90% confidence interval (CI) was 94.2%. The efficacy of the 2-day and the 1-day regimen was assumed ≥94%, providing reasonable precision given that the minimal acceptable efficacy for an MDA treatment is >90% [15]. Assuming 10% loss to follow-up, 100 participants per arm were needed to demonstrate ≥90% efficacy with 90% power.

### Statistical Analysis

**Figure 1.**
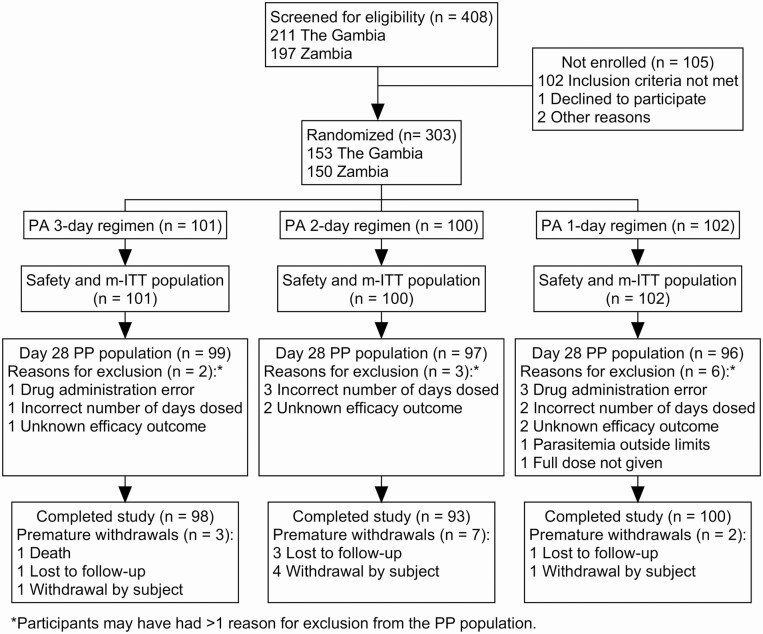
Participant disposition. Populations: safety population, all randomized participants who received at least 1 dose of study medication; m-ITT population, all randomized patients who received at least 1 treatment dose and who had confirmed positive parasitemia before treatment; PP population, all randomized patients who completed their treatment, had outcome data for the primary efficacy end point, and complied with the protocol. Abbreviations: mITT, microbiological-intention-to-treat; PA, pyronaridine-artesunate; PP, per-protocol.

For this exploratory study no formal statistical testing was planned. The primary efficacy endpoint was evaluated in the per-protocol (PP) population (Figure 1), with 1-sided (lower) 90% and 95% CI (Clopper–Pearson) calculated for each treatment arm. Two-sided exact 95% CI for the difference in day 28 APR between each pairwise comparison were calculated, that is, 3-day regimen versus 1-day regimen, 3-day regime versus 2-day regimen, and 2-day regimen versus 1-day regimen (Wilson method without continuity correction). Statistical analysis was performed using SAS version 9.4 or higher. A supportive analysis was conducted for the microbiological intention-to-treat (m-ITT) population (Figure 1).

Recrudescence rate and reinfection rate over 63 days were evaluated using Kaplan–Meier analysis in the m-ITT population. Participants with no recurrence event were censored at the last available parasite assessment date and those with major protocol deviations at the time of the protocol deviation. The proportion of parasite-free participants was determined for the PP population. Gametocyte carriage was determined as area under the gametocyte density–time curve (AUC) calculated according to the trapezoidal rule for all participants having at least 1 positive gametocyte count in the PP population.

## RESULTS

### Participants

Overall, 303 participants with confirmed *P. falciparum* monoinfection were enrolled (Figure 1). Baseline characteristics were generally comparable across the treatment arms (Table 2). Geometric mean parasite density was 573.9 µL^−1^, and 18.8% (55/292) of evaluable participants had baseline gametocytes detectable by microscopy.

**Table 2.** Baseline Characteristics

	Pyronaridine-artesunate Treatment Group			
Characteristics	3-day regimen (n = 101)	2-day regimen (n = 100)	1-day regimen (n = 102)	Overall (n = 303)
Country, n (%)				
The Gambia	51 (50.5)	50 (50.0)	52 (51.0)	153 (50.5)
Zambia	50 (49.5)	50 (50.0)	50 (49.0)	150 (49.5)
Sex, n (%)				
Male	60 (59.4)	39 (39.4)	49 (47.6)	148 (48.8)
Female	41 (40.6)	60 (60.6)	54 (52.4)	155 (51.2)
Age, y, mean (SD) [range]	15.0 (8.3) [6–48]	15.9 (9.9) [6–60]	16.6 (11.5) [6–64]	15.8 (10.0) [6–64]
Age group, n (%)				
5–≤12 y	48 (47.5)	49 (49.5)	48 (46.6)	145 (47.9)
>12–18 y	32 (31.7)	24 (24.2)	25 (24.3)	81 (26.7)
≥18 y	21 (20.8)	26 (26.3)	30 (29.1)	77 (25.4)
Weight by age group, kg, mean (SD) [range]				
5–≤12 y	29.0 (8.2) [20.7–65.2]	27.4 (6.3) [20.2–51.2]	26.1 (4.4) [20.6–40.1]	27.5 (6.6) [20.2–65.2]
>12–18 y	42.8 (7.5) [33.2–62.4]	46.5 (11.7) [27.8–72.0]	40.2 (6.7) [29.3–56.1]	43.1 (8.9) [27.8–72.0]
≥18 years	58.8 (11.6) [37.1–87.3]	56.7 (10.5) [44.4–94.0]	57.4 (10.7) [42.7–96.3]	57.5 (10.8) [37.1–96.3]
Asexual parasites, µL^−1^, geometric mean (range)	592.7 (20–38960)	579.6 (24–47600)	550.6 (16–33020)	573.9 (16–47600)
Participants with gametocytes, n/N (%)	17/99 (17.2)	20/97 (20.6)	18/96 (18.8)	55/292 (18.8)

### Efficacy

**Figure 2.**
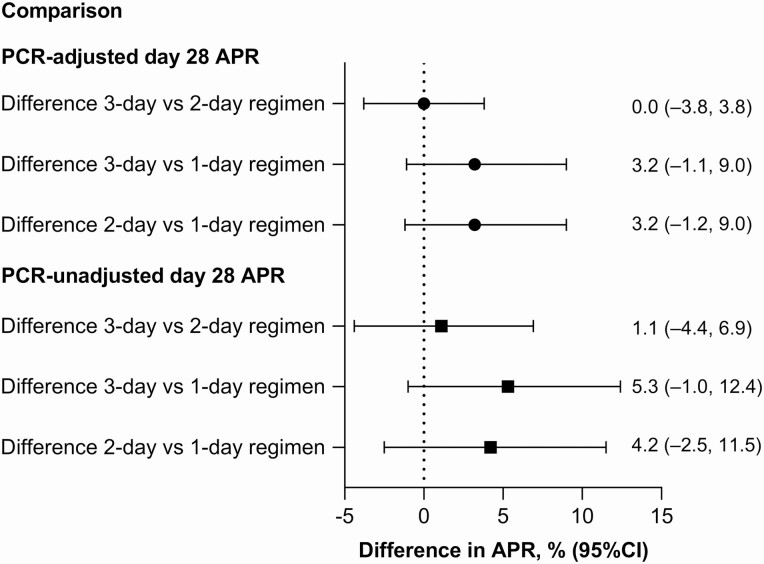
Adequate parasitological response at day 28 in the per-protocol population. Abbreviations: APR, adequate parasitological response; PCR, polymerase chain reaction.

**Figure 3.**
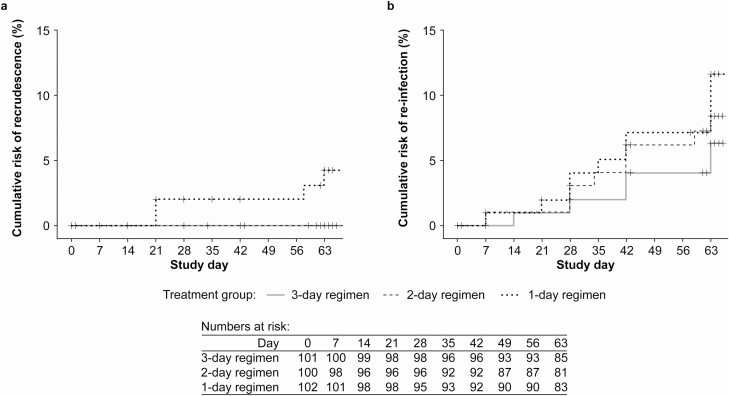
Kaplan-Meier estimates of (*a*) recrudescence; and (*b*) reinfection in the microbiological intention-to-treat population.

For the primary outcome, day 28 PCR-adjusted APR in the PP population was 100% (98/98) for the 3-day regimen, 100% (96/96) for the 2-day regimen, and 96.8% (91/94) for 1-day regimen; the lower limit of the 95% CI exceeded 90% for all regimens (Table 3). There was no significant difference in day 28 PCR-adjusted APR across the 3 study arms (Figure 2). Efficacy was maintained until day 63 for the 3-day and 2-day regimens but declined for the 1-day regimen (Table 3). The m-ITT analysis supported the primary analysis (Supplementary Table 1, Supplementary Figure 1). In the Kaplan-Meier analysis, there were no recrudescences through day 63 for the 3-day and 2-day regimens (Figure 3A). Reinfections were more frequent in the shorter treatment regimens (Figure 3B).

**Table 3.** Adequate Parasitological Response in the Per-protocol Population

	Pyronaridine-artesunate Treatment Group		
APR, n/N (%) [1-sided 95% CI]	3-day regimen (n = 99)	2-day regimen (n = 97)	1-day regimen (n = 96)^a^
PCR-adjusted			
Day 7	99/99 (100) [97.0]	97/97 (100) [97.0]	95/96 (99.0) [95.2]
Day 14	99/99 (100) [97.0]	96/96 (100) [96.9]	94/95 (98.9) [95.1]
Day 21	98/98 (100) [97.0]	96/96 (100) [96.9]	92/95 (96.8) [92.0]
Day 28	98/98 (100) [97.0]	96/96 (100) [96.9]	91/94 (96.8) [92.0]
Day 35	96/96 (100) [96.9]	93/93 (100) [96.8]	89/92 (96.7) [91.8]
Day 42	96/96 (100) [96.9]	92/92 (100) [96.8]	88/91 (96.7) [91.7]
Day 63	93/93 (100) [96.8]	86/86 (100) [96.6]	84/89 (94.4) [88.6]
PCR-unadjusted			
Day 7	99/99 (100) [97.0]	96/97 (99.0) [95.2]	94/96 (97.9) [93.6]
Day 14	98/99 (99.0) [95.3]	96/97 (99.0) [95.2]	94/96 (97.9) [93.6]
Day 21	98/99 (99.0) [95.3]	96/97 (99.0) [95.2]	91/96 (94.8) [89.4]
Day 28	97/99 (98.0) [93.8]	94/97 (96.9) [92.2]	89/96 (92.7) [86.7]
Day 35	96/98 (98.0) [93.7]	92/96 (95.8) [90.7]	88/96 (91.7) [85.5]
Day 42	94/98 (95.9) [90.9]	90/96 (93.8) [88.0]	86/96 (89.6) [83.0]
Day 63	91/97 (93.8) [88.2]	85/93 (91.4) [85.0]	81/96 (84.4) [77.0]

Abbreviations: APR, adequate parasitological response; CI, confidence interval; PCR, polymerase chain reaction.

^a^In the PCR-adjusted analysis, all treatment failures on or before day 42 and 4/5 on day 63 were late parasitological failures (parasitemia plus temperature <37°C), the remaining treatment failure on day 63 was a late clinical failure (parasitemia plus temperature ≥37°C).

**Figure 4.**
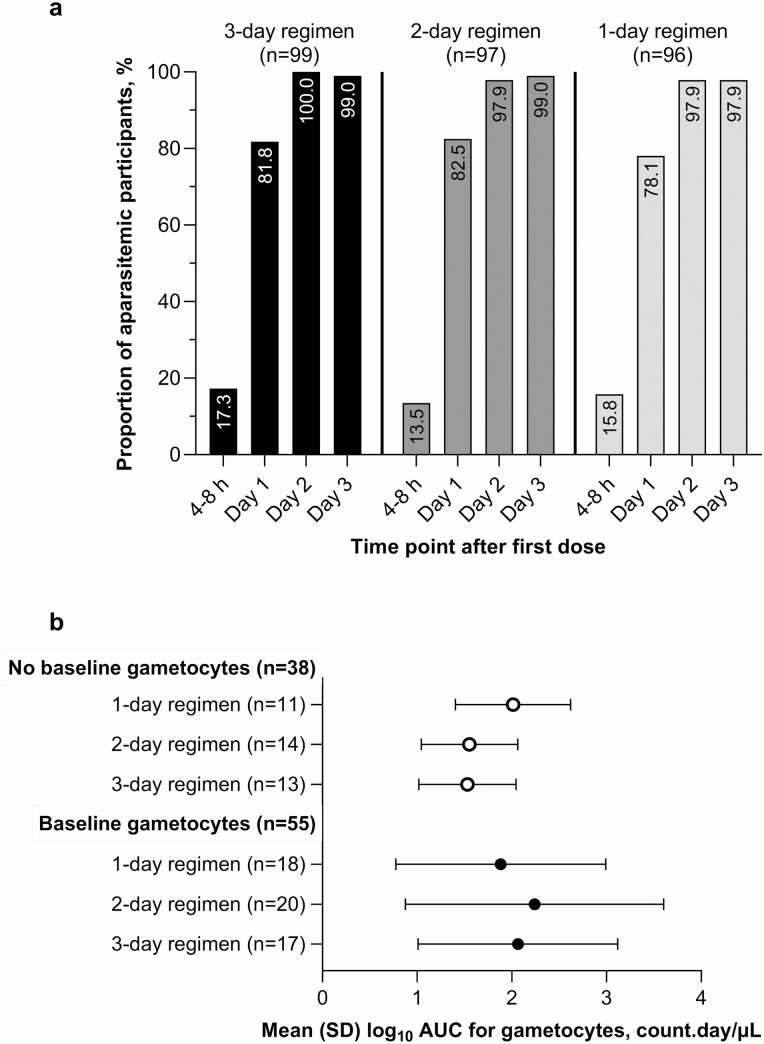
Parasite clearance in the per-protocol population: (*a*) proportion of participants with asexual parasite clearance until day 3; and (*b*) mean (SD) log_10_ area under the curve for gametocytes up to day 14 in participants with or without baseline gametocytes. Abbreviation: AUC, area under the gametocyte density–time curve.

The proportion of participants without infection as determined by microscopy between 4 and 8 hours post first PA dose and day 3 was similar for the 3 treatment groups (Figure 4A). The mean log_10_ AUC gametocytes until day 14 was similar for all 3 regimens (Figure 4B). However, all baseline gametocytes were cleared by day 21 with the 3-day regimen but persisted until day 28 with the 2-day and 1-day regimens, reappearing in 1 participant at day 63 with the day-1 regimen (Supplementary Table 2).

### Safety

**Figure 5.**
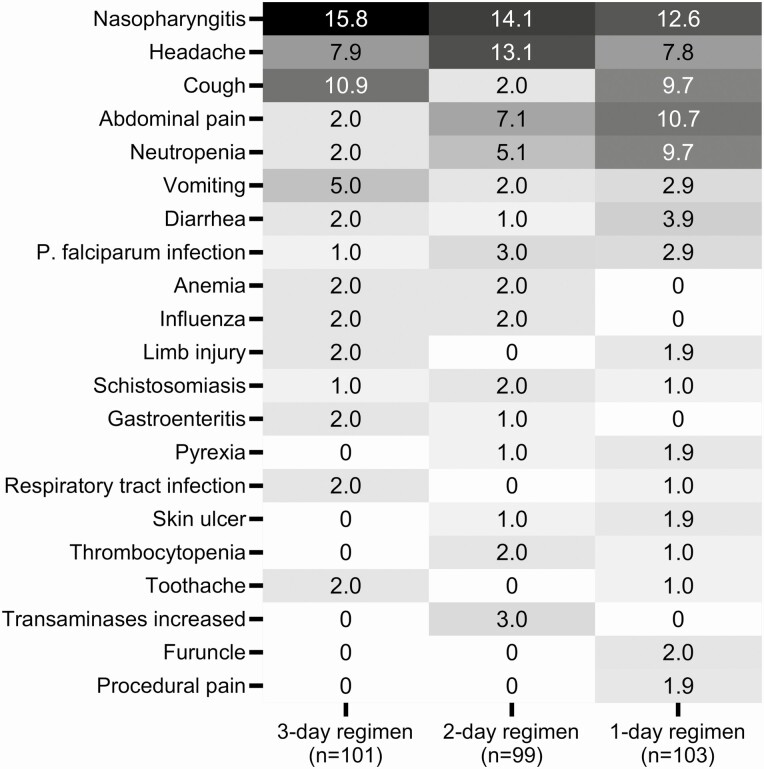
Most common treatment-emergent adverse events of any cause in the safety population. Adverse events occurring in >1 participant in any one treatment group. Values are percentage frequency. Participants may have had more than one adverse event. Abbreviation: P. falciparum, Plasmodium falciparum.

Adverse event frequency was similar between the 3-day (51.5% [52/101]), 2-day (52.5% [52/99]), and 1-day (54.4% [56/103]) regimens, although with some differences, that is, a lower incidence of cough with the 2-day regimen, and a higher incidence of neutropenia and abdominal pain with the 2-day and 1-day regimens versus the 3-day regimen (Figure 5). Most adverse events were grade 1 or 2 in severity (85% [136/160]); grade 3+ adverse events were more common in the 2-day (8.1% [8/99]) and 1-day (12.6% [13/103]) regimens versus the 3-day regimen (2.0% [2/101]) (Supplementary Table 3). The frequency of treatment-related adverse events was lower for the 3-day regimen (6.9% [7/101]) versus the 2-day (12.1% [12/99]) and 1-day (12.6% [13/103]) regimens (Supplementary Table 4), as was the frequency of malaria-related adverse events (2.0% [2/101], 6.1% [6/99], and 6.8% [7/103]), respectively) (Supplementary Table 5). There were 2 serious adverse events, 1 death of a 12-year-old male by drowning at day 30 (day-3 regimen), and a missed abortion in a 35-year-old female at day 149 resolved by a vacuum aspiration at day 152 (2-day regimen); neither was considered treatment related.

Most laboratory abnormalities were grade 1 or 2 and resolved by day 28 (Supplementary Tables 6 and 7). Post-baseline hemoglobin declines >2 g/dL were observed in 3.7% (11/297) of participants, but hemoglobin levels were >8 g/dL in all participants by day 28 (Table 4). Asymptomatic, transient increases in ALT/AST >3×ULN were observed in 6/301 (2.0%) participants, 3 of whom had increases >5×ULN. All values had normalized by day 28 (Table 4). There were no Hy’s Law cases.

**Table 4.** Changes in Hemoglobin, Alanine Aminotransferase and Aspartate Aminotransferase

		Pyronaridine-artesunate Treatment Group		
Parameter	Time Point	3-day regimen (n = 101)	2-day regimen (n = 99)	1-day regimen (n = 103)
Change in hemoglobin from baseline >2 g/dL, n/N (%)	Post-baseline	3/99 (3.0)	6/97 (6.2)	2/101 (2.0)
	Day 1	2/98 (2.0)	3/95 (3.2)	1/98 (1.0)
	Day 7	2/97 (2.1)	3/95 (3.2)	2/98 (2.0)
	Day 28	1/97 (1.0)	3/96 (3.1)	0/95
Mean hemoglobin (SD) [range], g/dL	Baseline	11.9 (1.5) [7.6–16.1]	12.1 (1.6) [7.3–17.4]	11.8 (1.6) [7.1–16.9]
	Day 1	11.6 (1.8) [7.2–19.2]	11.6 (1.5) [8.0–16.4]	11.5 (1.7) [6.9–19.0]
	Day 7	11.3 (1.4) [7.8–15.0]	11.6 (1.6) [8.5–19.9]	11.4 (1.8) [7.7–21.8]
	Day 28	12.0 (1.2) [8.2, 15.6]	12.2 (1.2) [9.1, 16.2]	12.0 (1.3) [8.6, 15.6]
Post-baseline ALT or AST >3×ULN, n/N (%)	Day 1	1/101 (1.0)	4/97 (4.1)	1/98 (1.0)
	Day 7	0/101	2/99 (2.0)	0/100
	Day 28	0/99	0/98	0/95
Post-baseline ALT or AST >5×ULN, n/N (%)	Day 1	0/101	2/97 (2.1)	1/98 (1.0)
	Day 7	0/101	0/99	0/100
	Day 28	0/99	0/98	0/95

Abbreviations: ALT, alanine aminotransferase; AST, aspartate aminotransferase; ×ULN, times the upper limit of normal.

## DISCUSSION

This study evaluated PA efficacy in asymptomatic individuals infected with *P. falciparum.* In addition, the potential consequences of poor adherence to the full 3-day regimen during MDA campaigns were evaluated by administration of 2-day and 1-day regimens. It is important to stress that this study was not designed to support any change to the 3-day PA regimen for the treatment of uncomplicated malaria, nor does it support abbreviated dosing to clear parasitemia in asymptomatic individuals. The reason for investigating incomplete treatment regimens was to determine PA efficacy when given for community-based interventions aiming at reducing the human reservoir of malaria infection, for example, MDA or mass testing and treatment. In these circumstances, when treatment may not be directly supervised, treated individuals may take only 1 or 2 days of treatment. Therefore, it is reassuring that the day-28 efficacy was similar across the 3 treatment regimens and that efficacy for the 3-day and 2-day regimens was maintained until day 63.

Single-dose PA had unexpectedly good efficacy in this population. In a murine blood-stage malaria model, single-dose pyronaridine was shown to reduce parasitemia more rapidly and completely than artesunate, chloroquine, or amodiaquine [35]. This potent effect may have been sufficient to suppress and/or clear parasites after only 1 dose in most individuals with low parasite density. Although there was no significant difference in PCR-adjusted day-28 APR, recrudescence occurred in the 1-day regimen group from day 7. Recrudescence drives resistance development [36]. Thus, there is a concern that the 1-day regimen would increase the risk or rapidity of resistance emergence to PA. In the Greater Mekong Sub-region, PA has been shown to be efficacious in regions where dihydroartemisinin-piperaquine and/or mefloquine-artesunate have been abandoned as first-line therapy for uncomplicated *P. falciparum* malaria owing to multi-drug resistance [21, 24–26]. Therefore, adherence to full treatment for PA is extremely important, given this combination might be an alternative option in case of emerging resistance to other ACTs [37]. With the 3-day and 2-day regimens, PCR-adjusted efficacy was maintained at 100% through day 63, with 1-sided 95% CIs exceeding 96% in both arms. Such a high efficacy probably reflects the low baseline parasite density (geometric mean 573.9, μL^−1^ blood); in contrast, African patients with uncomplicated malaria, have mean parasite densities typically above 15 000 μL^−1^ blood [20, 22, 27, 28, 30, 32].

Reinfections were more frequent with the 2-day and 1-day versus the 3-day PA regimen and occurred earlier; from day 7 with the 1-day and 2-day regimens versus day 14 for the 3-day regimen. This was expected given that a larger dose of pyronaridine will result in a longer half-life for the pyronaridine component, providing an extended period of post-treatment protection [30, 38]. Although the half-life of pyronaridine is about 14–18 days, the effect of this early difference in re-infection could still be observed at day 63.

Parasite clearance by day 3 was 99.0% for both the 3-day and 2-day regimens and slightly lower (97.9%) for the 1-day regimen. Similar rapid parasite clearance has been previously demonstrated for 3-day PA in patients with uncomplicated *P. falciparum* malaria [22, 29, 30, 32]. Only a small proportion of patients were parasitemic at day 3 following the 1-day PA regimen. However, because the half-life of artesunate and its active metabolite dihydroartemisinin is short (up to 1.5 hours) [39], these parasites will be exposed to pyronaridine monotherapy. As these parasites may be also those least susceptible to artesunate, any subsequent recrudescence increases the risk for the selection of artemisinin-resistant strains.

Clinical studies in patients with uncomplicated *P. falciparum* malaria indicate that ACTs have limited efficacy in clearing gametocytes, which is dependent primarily on the non-artemisinin component [40, 41]. Pyronaridine is thought to have limited efficacy against gametocytes, with conflicting *in vitro* data [42–45]. In Kenyan children with uncomplicated *P. falciparum* malaria treated with PA, quantitative reverse-transcription PCR indicated that 25.3% (20/79) of patients harbored gametocytes at day 14 [46]. In the current study, although the AUC values with all 3 regimens were similar, microscopically determined gametocytemia persisted to day 14 with the 3-day regimen, and to day 63 following the 1-day regimen. Thus, co-administration of PA and single low-dose primaquine may be needed if MDA is to rapidly clear gametocytes from asymptomatic individuals infected with falciparum malaria, as has been demonstrated with artemether-lumefantrine/primaquine and dihydroartemisinin-piperaquine/primaquine [14, 47, 48].

PA was generally well tolerated, with adverse events consistent with previous studies of 3-day treatment of patients with uncomplicated malaria [20, 22, 24–32, 49]. There was a trend for fewer adverse events with the 3-day versus the 2-day and 1-day regimens. Although the study population was asymptomatic for malaria, falciparum infection is not necessarily benign, being associated with immune system dysregulation and inflammation [50]. The full therapeutic dose may have been more effective in resolving the more subtle health impacts of malaria infection, and emergent malaria symptoms were observed more frequently with the abbreviated regimens. Consistent with the known safety profile for PA [20, 31, 32], transient, asymptomatic increases in ALT and AST were observed for 6 participants (2.0%). Notably, post-baseline ALT or AST >5×ULN only occurred with the 2-day and 1-day regimen.

A limitation of this study was the selection of participants based on microscopy, whereas individuals with sub-patent infection are an important component of the transmission reservoir [6, 7]. Nevertheless, given the lower parasite densities, PA efficacy is likely to be similar, if not higher against sub-microscopic infections. Moreover, we could not exclude the possibility of low-level residual parasitemia in PA-treated participants. In Kenyan children with uncomplicated *P. falciparum* malaria treated with either PA or artemether-lumefantrine, residual parasitemia at day 7 detected by quantitative PCR was not associated with parasite recurrence at day 28 or day 42 [51]. Given study participants were followed up until day 63 post-treatment in our study, it is unlikely that any recrudescence was missed. Nevertheless, it is possible that infections acquired during follow up may have had sub-patent densities at day 63 and may have been missed by microscopy. A further limitation of this study was the lack of an ACT comparator.

This study indicates the potential of PA for community-based malaria control interventions, in conjunction with other tools. The finding that the 2-day and 3-day regimens had similar efficacies in this population is reassuring given the challenges related to treatment adherence during MDA, as treatment is unlikely to be supervised for 3 days. However, this does not negate the importance of adherence to the 3-day regimen when used for acute malaria. This study supports further investigation of PA in comparative operational studies to examine adherence and outcomes in asymptomatic *P. falciparum* infection.

## Supplementary Material

ciab425_suppl_Supplementary_Appendix

## Supplementary Data

Supplementary materials are available at *Clinical Infectious Diseases* online. Consisting of data provided by the authors to benefit the reader, the posted materials are not copyedited and are the sole responsibility of the authors, so questions or comments should be addressed to the corresponding author.

## Notes

***Acknowledgments.*** The authors thank the study population and local staff, who made this study possible. They thank the following individuals for their assistance during the trial: Sampa Sanneh in The Gambia and Eric Njunju, Patrick Tshilab, Samson Mwale, Phidelis Malunga, Geoffrey Mwanza, Susan Ayvaliklis, Joyce W. Mulenga, Diffie Gondwe, Kelvin Kaela, Friday Mukuluka, Proscovia Banda, Harry Kachesa, Priscilla Ng’andu, Maureen Mwape and Harriet Bwembya and Sr. Catherine Tente, St. Paul’s Hospital administrator in Zambia. They would also like to acknowledge the contribution of Sherry Armstrong-Wilkinson, the Clinical trial manager. Naomi Richardson of Magenta Communications Ltd provided editorial and graphic services and was funded by Medicines for Malaria Venture. After publication, trial data will be made available on request to the corresponding author. De-identified participant data will be provided after approval by the sponsor and trial management group.

***Financial support.*** This work was supported by Shin Poong Pharmaceutical and Medicines for Malaria Venture.

***Potential conflicts of interest.*** I. B. F. and S. D. are full-time employees of Medicines for Malaria Venture, and J. S. is employed by Shin Poong Pharmaceutical Company, Ltd., and is an employee of Medicines for Malaria Venture. R. M. consulted for Shin Poong during the study and is the Shin Poong qualified person for pharmacovigilance; R. M. is a full-time employee at Artemida Pharma Limited and is responsible for safety aspects of pyronaridine-artesunate on behalf of Shin Poong. S. A. B. is a full-time employee at Artemida Pharma Limited and responsible for project oversight for pyronaridine-artesunate. All other authors report no potential conflicts.All authors have submitted the ICMJE Form for Disclosure of Potential Conflicts of Interest. Conflicts that the editors consider relevant to the content of the manuscript have been disclosed.
