# Supplementary material for: Efficacy, Safety and Tolerability of Pyronaridine-artesunate in Asymptomatic Malaria-infected Individuals: a Randomized Controlled Trial
Source: Clin Infect Dis. 2021 May 13;74(2):180–8. doi: 10.1093/cid/ciab425 (PMC8800175; doi:10.1093/cid/ciab425)
Supplement: ciab425_suppl_Supplementary_Appendix [file ciab425_suppl_supplementary_appendix.docx]

**SUPPLEMENTARY MATERIAL**

**Efficacy, Safety and Tolerability of Pyronaridine-artesunate in Asymptomatic Malaria-infected Individuals: a Randomized Controlled Trial**

Dabira, et al.

**Contents**

Supplementary Methods 1. Study Eligibility Criteria 2

Supplementary Table 1. Adequate parasitological response in the modified intention-to-treat population. 3

Supplementary Figure 1. Adequate parasitological response at day 28 in the modified intention-to-treat population. 4

Supplementary Table 2. Participants with *Plasmodium falciparum* gametocytes (per-protocol population). 5

Supplementary Table 3. All treatment-emergent adverse events of any cause by severity (safety population). 6

Supplementary Table 4. Treatment-emergent adverse events considered to be study drug related (safety population). 10

Supplementary Table 5. Treatment-emergent adverse events considered to be related to malaria (safety population). 11

Supplementary Table 6. Hematology. 12

Supplementary Table 7. Clinical biochemistry. 15

Supplementary Methods 1. Study Eligibility Criteria

**Inclusion Criteria**

1. Evidence of asymptomatic infection with *Plasmodium falciparum* mono-infection on thin and thick blood smears with parasite density between 20/μL and 50,000/μL;

2. Absence of any clinical symptoms of malaria at the time of enrolment and within 72 hours before enrolment;

3. Age >5 years old and >20 kg body weight;

4. Ability to swallow oral medication;

5. Evidence of a personally signed and dated Informed Consent document indicating that the participant (or a legally acceptable representative if a participant is <18 years of age) has been informed of all pertinent aspects of the study and that all questions by the participant have been sufficiently answered. Assent will be obtained from participants <18 years of age as required by national regulations and

6. Participants who are willing to and are able to comply with scheduled visits, treatment plan, laboratory tests, and other study procedures.

**Exclusion Criteria**

Participants presenting with any of the following will not be included in the study:

1. Haemoglobin <7 g/dL (measured at screening);

2. History of having received any antimalarial treatment (alone or in combination) during the following periods before screening:

a. Piperaquine, mefloquine, naphthoquine or sulfadoxine-pyrimethamine within 6 weeks prior to screening

b. Amodiaquine, chloroquine within 4 weeks prior to screening

c. Any artemisinin derivative (artesunate, artemether or dihydroartemisinin), quinine, lumefantrine or any other anti-malarial treatment or antibiotic with antimalarial activity (including cotrimoxazole, tetracyclines, quinolones and fluoroquinolones and azithromycin) within 14 days prior to screening;

3. Any herbal products or traditional medicines during the 7 days prior to screening (if spontaneously reported by the patient);

4. Known allergy to the study drugs (pyronaridine and/or any artemisinin derivatives);

5. Positive urinary pregnancy test for women of reproductive age;

6. Lactating women;

7. Evidence of severe malnutrition, defined as follows for the specified age-groups:

a. Participants aged ≥20 years: BMI <16 kg/m^2^

b. Participants aged <20 years: BMI-for-age z-score <-3

8. Participation in other studies within 30 days before the current study begins and/or during study participation;

9. Inability to comprehend and/or unwillingness to follow the study protocol;

10. Previously randomized in this study;

11. Severe acute or chronic medical or psychiatric condition or laboratory abnormality that may increase the risk associated with study participation or investigational product administration or may interfere with the interpretation of study results and, in the judgment of the investigator, would make the participant inappropriate for entry into this study. Examples would include but not limited to: a. Immunological disorders (including known seropositive HIV antibody),

b. Severe psychiatric disorders (active depression, recent history of depression, generalised anxiety, psychosis, schizophrenia or other major psychiatric disorders) and major medical disorders related to cardiovascular, respiratory (including active tuberculosis), renal, gastrointestinal, endocrine, infectious, malignancy, neurological (including auditory) and history of convulsions or other abnormality (including recent head trauma),

c. Clinical signs or symptoms of hepatic injury (such as nausea, abdominal pain associated with jaundice) or known severe liver disease (i.e. decompensated cirrhosis, Child-Pugh stage 3 or 4)

12. Participant the Investigator considers at particular risk of receiving an anti-malarial or of participating in the study

Supplementary Table 1. Adequate parasitological response in the modified intention-to-treat population.

| APR, n/N (%) [one sided 95% CI] | Pyronaridine-artesunate treatment group | | |
| --- | --- | --- | --- |
|  | 3-day regimen  (n = 101) | 2-day regimen  (n = 100) | 1-day regimen  (n = 102) |
| PCR-adjusted |  |  |  |
| Day 7 | 101/101 (100) [97.1] | 98/100 (98.0) [93.8] | 100/102 (98.0) [94.0] |
| Day 14 | 100/101 (99.0) [95.4] | 69/100 (96.0) [91.1] | 98/102 (96.1) [91.3] |
| Day 21 | 99/101 (98.0) [93.9] | 96/100 (96.0) [91.1] | 96/102 (94.1) [88.7] |
| Day 28 | 99/101 (98.0) [93.9] | 96/100 (96.0) [91.1] | 95/102 (93.1) [87.5] |
| Day 35 | 97/101 (96.0) [91.2] | 93/100 (93.0) [87.3] | 93/102 (91.2) [85.1] |
| Day 42 | 97/101 (96.0) [91.2] | 92/100 (92.0) [86.0] | 92/102 (90.2) [83.9] |
| Day 63 | 94/101 (93.1) [87.4] | 86/100 (86.0) [79.0] | 88/102 (86.3) [79.4] |
| PCR-unadjusted |  |  |  |
| Day 7 | 101/101 (100) [97.1] | 97/100 (97.0) [92.4] | 99/102 (97.1) [92.6] |
| Day 14 | 99/101 (98.0) [93.9] | 96/100 (96.0) [91.1] | 98/102 (96.1) [91.3] |
| Day 21 | 99/101 (98.0) [93.9] | 96/100 (96.0) [91.1] | 95/102 (93.1) [87.5] |
| Day 28 | 98/101 (97.0) [92.5] | 94/100 (94.0) [88.5] | 93/102 (91.2) [85.1] |
| Day 35 | 97/101 (96.0) [91.2] | 92/100 (92.0) [86.0] | 92/102 (90.2) [83.9] |
| Day 42 | 95/101 (94.1) [88.6] | 90/100 (90.0) [83.6] | 90/102 (88.2) [81.6] |
| Day 63 | 92/101 (91.1) [85.0] | 85/100 (85.0) [77.8] | 84/102 (82.4) [75.0] |

Abbreviations: PCR, polymerase chain reaction; APR, adequate parasitological response.

Supplementary Figure 1. Adequate parasitological response at day 28 in the modified intention-to-treat population.


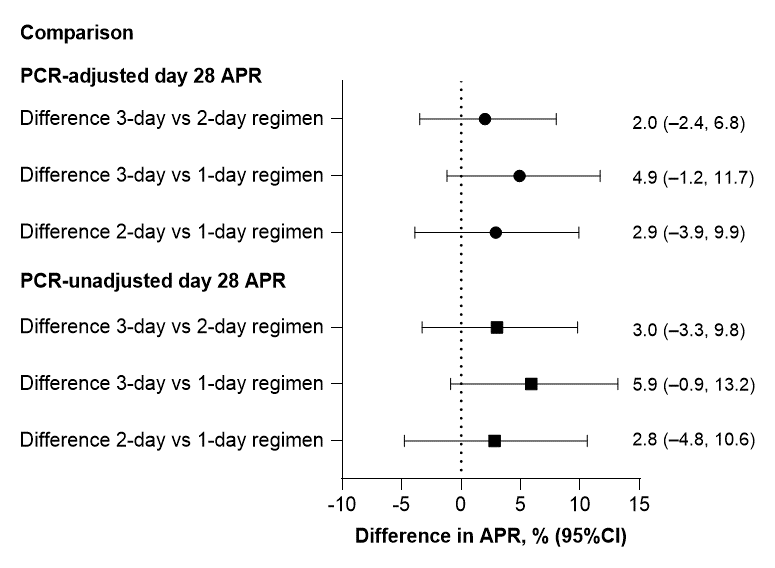


Abbreviations: APR, adequate parasitological response.

Supplementary Table 2. Participants with *Plasmodium falciparum* gametocytes (per-protocol population).

| Study time point | Pyronaridine-artesunate treatment group | | |
| --- | --- | --- | --- |
|  | 3-day regimen  (n = 99) | 2-day regimen  (n = 97) | 1-day regimen  (n = 96) |
| Baseline | 17/99 (17.2) | 20/97 (20.6) | 18/96 (18.8) |
| 4–8 h post first dose | 15/98 (15.3) | 16/96 (16.7) | 14/95 (14.7) |
| Day 1 | 13/99 (13.1) | 15/97 (15.5) | 15/96 (15.6) |
| Day 2 | 9/99 (9.1) | 15/97 (15.5) | 12/96 (12.5) |
| Day 3 | 6/99 (6.1) | 11/97 (11.3) | 13/95 (13.7) |
| Day 7 | 10/99 (10.1) | 11/96 (11.5) | 8/96 (8.3) |
| Day 14 | 2/98 (2.0) | 8/96 (8.3) | 3/94 (3.2) |
| Day 21 | 0/98 | 3/96 (3.1) | 1/93 (1.1) |
| Day 28 | 0/98 | 3/96 (3.1) | 2/90 (2.2) |
| Day 35 | 0/98 | 0/91 | 0/88 |
| Day 42 | 0/95 | 0/92 | 0/88 |
| Day 63 | 0/93 | 0/87 | 1/86 (1.2) |

Values are n/N (%) for patients with baseline gametocytes.

Supplementary Table 3. All treatment-emergent adverse events of any cause by severity (safety population).

| Adverse event, n (%) | Severity grade | Pyronaridine-artesunate treatment group | | | Overall  (n = 303) |
| --- | --- | --- | --- | --- | --- |
|  |  | 3-day regimen  (n = 101) | 2-day regimen  (n = 99) | 1-day regimen  (n = 103) |  |
| At least one adverse event | Any | 52 (51.5) | 52 (52.5) | 56 (54.4) | 160 (52.8) |
|  | 1 | 13 (12.9) | 20 (20.2) | 14 (13.6) | 47 (15.5) |
|  | 2 | 36 (35.6) | 24 (24.2) | 29 (28.2) | 89 (29.4) |
|  | 3 | 2 (2.0) | 8 (8.1) | 13 (12.6) | 23 (7.6) |
|  | 4 | 0 | 0 | 0 | 0 |
|  | 5 | 1 (1.0) | 0 | 0 | 1 (0.3) |
| Blood and lymphatic system disorders |  |  |  |  |  |
| Anemia | Any | 2 (2.0) | 2 (2.0) | 0 | 4 (1.3) |
|  | 1 | 0 | 1 (1.0) | 0 | 1 (0.3) |
|  | 2 | 2 (2.0) | 1 (1.0) | 0 | 3 (1.0) |
| Leukocytosis | Any | 1 (1.0) | 1 (1.0) | 0 | 2 (0.7) |
|  | 1 | 1 (1.0) | 1 (1.0) | 0 | 2 (0.7) |
| Neutropenia | Any | 2 (2.0) | 5 (5.1) | 10 (9.7) | 17 (5.6) |
|  | 3 | 2 (2.0) | 5 (5.1) | 10 (9.7) | 17 (5.6) |
| Thrombocytopenia | Any | 0 | 2 (2.0) | 1 (1.0) | 3 (1.0) |
|  | 1 | 0 | 1 (1.0) | 1 (1.0) | 2 (0.7) |
|  | 2 | 0 | 1 (1.0) | 0 | 1 (0.3) |
| Gastrointestinal disorders |  |  |  |  |  |
| Abdominal pain | Any | 2 (2.0) | 7 (7.1) | 11 (10.7) | 20 (6.6) |
|  | 1 | 1 (1.0) | 6 (6.1) | 8 (7.8) | 15 (5.0) |
|  | 2 | 1 (1.0) | 1 (1.0) | 3 (2.9) | 5 (1.7) |
| Diarrhea | Any | 2 (2.0) | 1 (1.0) | 4 (3.9) | 7 (2.3) |
|  | 1 | 2 (2.0) | 1 (1.0) | 2 (1.9) | 5 (1.7) |
|  | 2 | 0 | 0 | 2 (1.9) | 2 (0.7) |
| Nausea | Any | 0 | 1 (1.0) | 0 | 1 (0.3) |
|  | 1 | 0 | 1 (1.0) | 0 | 1 (0.3) |
| Toothache | Any | 2 (2.0) | 0 | 1 (1.0) | 3 (1.0) |
|  | 2 | 2 (2.0) | 0 | 1 (1.0) | 3 (1.0) |
| Vomiting | Any | 5 (5.0) | 2 (2.0) | 3 (2.9) | 10 (3.3) |
|  | 1 | 4 (4.0) | 2 (2.0) | 3 (2.9) | 9 (3.0) |
|  | 2 | 1 (1.0) | 0 | 0 | 1 (0.3) |
| General disorders and administration site conditions |  |  |  |  |  |
| Drowning | Any | 1 (1.0) | 0 | 0 | 1 (0.3) |
|  | 5 | 1 (1.0) | 0 | 0 | 1 (0.3) |
| Pain | Any | 0 | 0 | 1 (1.0) | 1 (0.3) |
|  | 1 | 0 | 0 | 1 (1.0) | 1 (0.3) |
| Pyrexia | Any | 0 | 1 (1.0) | 2 (1.9) | 3 (1.0) |
|  | 1 | 0 | 1 (1.0) | 1 (1.0) | 2 (0.7) |
|  | 2 | 0 | 0 | 1 (1.0) | 1 (0.3) |
| Infections and infestations |  |  |  |  |  |
| Abscess limb | Any | 0 | 0 | 1 (1.0) | 1 (0.3) |
|  | 2 | 0 | 0 | 1 (1.0) | 1 (0.3) |
| Body tinea | Any | 0 | 0 | 1 (1.0) | 1 (0.3) |
|  | 1 | 0 | 0 | 1 (1.0) | 1 (0.3) |
| Bronchitis | Any | 0 | 1 (1.0) | 0 | 1 (0.3) |
|  | 2 | 0 | 1 (1.0) | 0 | 1 (0.3) |
| Conjunctivitis | Any | 0 | 0 | 1 (1.0) | 1 (0.3) |
|  | 1 | 0 | 0 | 1 (1.0) | 1 (0.3) |
| Furuncle | Any | 0 | 0 | 2 (1.9) | 2 (0.7) |
|  | 2 | 0 | 0 | 2 (1.9) | 2 (0.7) |
| Gastroenteritis | Any | 2 (2.0) | 1 (1.0) | 0 | 3 (1.0) |
|  | 2 | 2 (2.0) | 1 (1.0) | 0 | 3 (1.0) |
| Hepatitis B | Any | 0 | 1 (1.0) | 0 | 1 (0.3) |
|  | 1 | 0 | 1 (1.0) | 0 | 1 (0.3) |
| Influenza | Any | 2 (2.0) | 2 (2.0) | 0 | 4 (1.3) |
|  | 2 | 2 (2.0) | 2 (2.0) | 0 | 4 (1.3) |
| Nasopharyngitis | Any | 16 (15.8) | 14 (14.1) | 13 (12.6) | 43 (14.2) |
|  | 1 | 3 (3.0) | 1 (1.0) | 3 (2.9) | 7 (2.3) |
|  | 2 | 13 (12.9) | 13 (13.1) | 10 (9.7) | 36 (11.9) |
| *P. falciparum* infection | Any | 1 (1.0) | 3 (3.0) | 3 (2.9) | 7 (2.3) |
|  | 1 | 0 | 1 (1.0) | 0 | 1 (0.3) |
|  | 2 | 1 (1.0) | 2 (2.0) | 3 (2.9) | 6 (2.0) |
| Pneumonia | Any | 1 (1.0) | 0 | 0 | 1 (0.3) |
|  | 2 | 1 (1.0) | 0 | 0 | 1 (0.3) |
| Respiratory tract infection | Any | 2 (2.0) | 0 | 1 (1.0) | 3 (1.0) |
|  | 2 | 2 (2.0) | 0 | 1 (1.0) | 3 (1.0) |
| Schistosomiasis | Any | 1 (1.0) | 2 (2.0) | 1 (1.0) | 4 (1.3) |
|  | 1 | 1 (1.0) | 1 (1.0) | 0 | 2 (0.7) |
|  | 2 | 0 | 1 (1.0) | 1 (1.0) | 2 (0.7) |
| Tonsillitis | Any | 0 | 1 (1.0) | 1 (1.0) | 2 (0.7) |
|  | 2 | 0 | 1 (1.0) | 1 (1.0) | 2 (0.7) |
| Upper respiratory tract infection | Any | 0 | 1 (1.0) | 0 | 1 (0.3) |
|  | 2 | 0 | 1 (1.0) | 0 | 1 (0.3) |
| Urinary tract infection | Any | 0 | 1 (1.0) | 1 (1.0) | 2 (0.7) |
|  | 1 | 0 | 0 | 1 (1.0) | 1 (0.3) |
|  | 2 | 0 | 1 (1.0) | 0 | 1 (0.3) |
| Varicella | Any | 1 (1.0) | 0 | 0 | 1 (0.3) |
|  | 1 | 1 (1.0) | 0 | 0 | 1 (0.3) |
| Wound infection | Any | 0 | 0 | 2 (1.9) | 2 (0.7) |
|  | 2 | 0 | 0 | 1 (1.0) | 1 (0.3) |
|  | 3 | 0 | 0 | 1 (1.0) | 1 (0.3) |
| Injury, poisoning and procedural complications |  |  |  |  |  |
| Contusion | Any | 0 | 1 (1.0) | 0 | 1 (0.3) |
|  | 1 | 0 | 1 (1.0) | 0 | 1 (0.3) |
| Limb injury | Any | 2 (2.0) | 0 | 2 (1.9) | 4 (1.3) |
|  | 1 | 2 (2.0) | 0 | 1 (1.0) | 3 (1.0) |
|  | 2 | 0 | 0 | 1 (1.0) | 1 (0.3) |
| Lip injury | Any | 0 | 1 (1.0) | 0 | 1 (0.3) |
|  | 1 | 0 | 1 (1.0) | 0 | 1 (0.3) |
| Procedural pain | Any | 0 | 0 | 2 (1.9) | 2 (0.7) |
|  | 2 | 0 | 0 | 2 (1.9) | 2 (0.7) |
| Soft tissue injury | Any | 1 (1.0) | 0 | 0 | 1 (0.3) |
|  | 2 | 1 (1.0) | 0 | 0 | 1 (0.3) |
| Wound | Any | 1 (1.0) | 1 (1.0) | 0 | 2 (0.7) |
|  | 1 | 0 | 1 (1.0) | 0 | 1 (0.3) |
|  | 2 | 1 (1.0) | 0 | 0 | 1 (0.3) |
| Investigations |  |  |  |  |  |
| Alanine aminotransferase increased | Any | 0 | 1 (1.0) | 1 (1.0) | 2 (0.7) |
|  | 1 | 0 | 1 (1.0) | 1 (1.0) | 2 (0.7) |
| Aspartate aminotransferase increased | Any | 1 (1.0) | 0 | 0 | 1 (0.3) |
|  | 2 | 1 (1.0) | 0 | 0 | 1 (0.3) |
| Blood bilirubin increased | Any | 0 | 0 | 1 (1.0) | 1 (0.3) |
|  | 1 | 0 | 0 | 1 (1.0) | 1 (0.3) |
| Hemoglobin decreased | Any | 0 | 0 | 1 (1.0) | 1 (0.3) |
|  | 2 | 0 | 0 | 1 (1.0) | 1 (0.3) |
| Hepatic enzyme increased | Any | 0 | 1 (1.0) | 1 (1.0) | 2 (0.7) |
|  | 3 | 0 | 1 (1.0) | 1 (1.0) | 2 (0.7) |
| Neutrophil count decreased | Any | 0 | 1 (1.0) | 1 (1.0) | 2 (0.7) |
|  | 3 | 0 | 1 (1.0) | 1 (1.0) | 2 (0.7) |
| Platelet count decreased | Any | 0 | 0 | 1 (1.0) | 1 (0.3) |
|  | 3 | 0 | 0 | 1 (1.0) | 1 (0.3) |
| Transaminase increased | Any | 0 | 3 (3.0) | 0 | 3 (1.0) |
|  | 2 | 0 | 2 (2.0) | 0 | 2 (0.7) |
|  | 3 | 0 | 1 (1.0) | 0 | 1 (0.3) |
| Musculoskeletal and connective tissue disorders |  |  |  |  |  |
| Back pain | Any | 0 | 1 (1.0) | 0 | 1 (0.3) |
|  | 2 | 0 | 1 (1.0) | 0 | 1 (0.3) |
| Musculoskeletal pain | Any | 0 | 1 (1.0) | 0 | 1 (0.3) |
|  | 1 | 0 | 1 (1.0) | 0 | 1 (0.3) |
| Neck pain | Any | 1 (1.0) | 0 | 0 | 1 (0.3) |
|  | 2 | 1 (1.0) | 0 | 0 | 1 (0.3) |
| Nervous system disorders |  |  |  |  |  |
| Headache | Any | 8 (7.9) | 13 (13.1) | 8 (7.8) | 29 (9.6) |
|  | 1 | 3 (3.0) | 9 (9.1) | 4 (3.9) | 16 (5.3) |
|  | 2 | 5 (5.0) | 4 (4.0) | 4 (3.9) | 13 (4.3) |
| Pregnancy, puerperium and perinatal conditions |  |  |  |  |  |
| Abortion missed | Any | 0 | 1 (1.0) | 0 | 1 (0.3) |
|  | 1 | 0 | 1 (1.0) | 0 | 1 (0.3) |
| Reproductive system and breast disorders |  |  |  |  |  |
| Dysmenorrhea | Any | 1 (1.0) | 0 | 0 | 1 (0.3) |
|  | 2 | 1 (1.0) | 0 | 0 | 1 (0.3) |
| Vaginal discharge | Any | 0 | 0 | 1 (1.0) | 1 (0.3) |
|  | 1 | 0 | 0 | 1 (1.0) | 1 (0.3) |
| Respiratory, thoracic and mediastinal disorders |  |  |  |  |  |
| Cough | Any | 11 (10.9) | 2 (2.0) | 10 (9.7) | 23 (7.6) |
|  | 1 | 5 (5.0) | 2 (2.0) | 7 (6.8) | 14 (4.6) |
|  | 2 | 6 (5.9) | 0 | 3 (2.9) | 9 (3.0) |
| Epistaxis | Any | 0 | 0 | 1 (1.0) | 1 (0.3) |
|  | 2 | 0 | 0 | 1 (1.0) | 1 (0.3) |
| Oropharyngeal pain | Any | 1 (1.0) | 1 (1.0) | 0 | 2 (0.7) |
|  | 1 | 1 (1.0) | 1 (1.0) | 0 | 2 (0.7) |
| Sneezing | Any | 0 | 1 (1.0) | 1 (1.0) | 2 (0.7) |
|  | 1 | 0 | 0 | 1 (1.0) | 1 (0.3) |
|  | 2 | 0 | 1 (1.0) | 0 | 1 (0.3) |
| Skin and subcutaneous tissue disorders |  |  |  |  |  |
| Rash | Any | 0 | 1 (1.0) | 1 (1.0) | 2 (0.7) |
|  | 1 | 0 | 1 (1.0) | 0 | 1 (0.3) |
|  | 2 | 0 | 0 | 1 (1.0) | 1 (0.3) |
| Skin mass | Any | 0 | 0 | 1 (1.0) | 1 (0.3) |
|  | 1 | 0 | 0 | 1 (1.0) | 1 (0.3) |
| Skin ulcer | Any | 0 | 1 (1.0) | 2 (1.9) | 3 (1.0) |
|  | 1 | 0 | 1 (1.0) | 1 (1.0) | 2 (0.7) |
|  | 2 | 0 | 0 | 1 (1.0) | 1 (0.3) |
| Vascular disorders |  |  |  |  |  |
| Hypertension | Any | 0 | 0 | 1 (1.0) | 1 (0.3) |
|  | 2 | 0 | 0 | 1 (1.0) | 1 (0.3) |
| Hypotension | Any | 1 (1.0) | 0 | 0 | 1 (0.3) |
|  | 2 | 1 (1.0) | 0 | 0 | 1 (0.3) |

Participants may have had more than one adverse event.

Grades are based on the Common Terminology Criteria for Adverse Events (CTCAE) v5.0.

Supplementary Table 4. Treatment-emergent adverse events considered to be study drug related (safety population).

| Adverse event, n (%) | Pyronaridine-artesunate treatment group | | | Overall  (n = 303) |
| --- | --- | --- | --- | --- |
|  | 3-day regimen  (n = 101) | 2-day regimen  (n = 99) | 1-day regimen  (n = 103) |  |
| At least one adverse event | 7 (6.9) | 12 (12.1) | 13 (12.6) | 32 (10.6) |
| Anemia | 1 (1.0) | 2 (2.0) | 0 | 3 (1.0) |
| Neutropenia | 0 | 0 | 1 (1.0) | 1 (0.3) |
| Vomiting | 4 (4.0) | 2 (2.0) | 3 (2.9) | 9 (3.0) |
| Abdominal pain | 1 (1.0) | 3 (3.0) | 4 (3.9) | 8 (2.6) |
| Diarrhea | 0 | 0 | 1 (1.0) | 1 (0.3) |
| Nausea | 0 | 1 (1.0) | 0 | 1 (0.3) |
| Transaminases increased | 0 | 3 (3.0) | 0 | 3 (1.0) |
| Alanine aminotransferase increased | 0 | 1 (1.0) | 1 (1.0) | 2 (0.7) |
| Hepatic enzymes increased | 0 | 1 (1.0) | 1 (1.0) | 2 (0.7) |
| Aspartate aminotransferase increased | 1 (1.0) | 0 | 0 | 1 (0.3) |
| Blood bilirubin increased | 0 | 0 | 1 (1.0) | 1 (0.3) |
| Hemoglobin decreased | 0 | 0 | 1 (1.0) | 1 (0.3) |
| Neutrophil count decreased | 0 | 0 | 1 (1.0) | 1 (0.3) |
| Platelet count decreased | 0 | 0 | 1 (1.0) | 1 (0.3) |
| Headache | 0 | 1 (1.0) | 0 | 1 (0.3) |

Participants may have had more than one adverse event.

Supplementary Table 5. Treatment-emergent adverse events considered to be related to malaria (safety population).

| Adverse event, n (%) | Pyronaridine-artesunate treatment group | | | Overall  (n = 303) |
| --- | --- | --- | --- | --- |
|  | 3-day regimen  (n = 101) | 2-day regimen  (n = 99) | 1-day regimen  (n = 103) |  |
| At least one adverse event | 2 (2.0) | 6 (6.1) | 7 (6.8) | 15 (5.0) |
| Anemia | 1 (1.0) | 2 (2.0) | 0 | 3 (1.0) |
| Thrombocytopenia | 0 | 1 (1.0) | 1 (1.0) | 2 (0.7) |
| Pain | 0 | 0 | 1 (1.0) | 1 (0.3) |
| Pyrexia | 0 | 0 | 1 (1.0) | 1 (0.3) |
| *P. falciparum* infection | 1 (1.0) | 3 (3.0) | 3 (2.9) | 7 (2.3) |
| Hemoglobin decreased | 0 | 0 | 1 (1.0) | 1 (0.3) |
| Platelet count decreased | 0 | 0 | 1 (1.0) | 1 (0.3) |

Participants may have had more than one adverse event.

Supplementary Table 6. Hematology.

| Parameter | Time point | Grade | Pyronaridine-artesunate treatment group | | |
| --- | --- | --- | --- | --- | --- |
|  |  |  | 3-day regimen (n=101) | 2-day regimen (n=99) | 1-day regimen (n=103) |
| Hemoglobin, g/dL (decrease) | Baseline | 0 | 52 (51.5) | 62 (62.6) | 44 (42.7) |
|  |  | 1 | 37 (36.6) | 28 (28.3) | 49 (47.6) |
|  |  | 2 | 8 (7.9) | 6 (6.1) | 9 (8.7) |
|  |  | 3 | 2 (2.0) | 1 (1.0) | 1 (1.0) |
|  |  | Missing | 2 (2.0) | 2 (2.0) | 0 |
|  | Day 1 | 0 | 35 (34.7) | 42 (43.4) | 33 (32.0) |
|  |  | 1 | 54 (53.5) | 43 (43.4) | 50 (48.5) |
|  |  | 2 | 6 (5.9) | 10 (10.1) | 14 (13.6) |
|  |  | 3 | 3 (3.0) | 0 | 1 (1.0) |
|  |  | Missing | 3 (3.0) | 4 (4.0) | 5 (4.9) |
|  | Day 7 | 0 | 32 (31.7) | 41 (41.4) | 32 (31.1) |
|  |  | 1 | 51 (50.5) | 45 (45.5) | 53 (51.5) |
|  |  | 2 | 14 (13.9) | 11 (11.1) | 12 (11.7) |
|  |  | 3 | 2 (2.0) | 0 | 1 (1.0) |
|  |  | Missing | 2 (2.0) | 2 (2.0) | 5 (4.9) |
|  | Day 28 | 0 | 56 (55.4) | 62 (62.6) | 52 (50.5) |
|  |  | 1 | 39 (38.6) | 35 (35.4) | 40 (38.8) |
|  |  | 2 | 4 (4.0) | 1 (1.0) | 3 (2.9) |
|  |  | Missing | 2 (2.0) | 1 (1.0) | 8 (7.8) |
| Platelets, 10^9^/L (decrease) | Baseline | 0 | 80 (79.2) | 86 (86.9) | 93 (90.3) |
|  |  | 1 | 19 (18.8) | 10 (10.1) | 10 (9.7) |
|  |  | 2 | 0 | 1 (1.0) | 0 |
|  |  | Missing | 2 (2.0) | 2 (2.0) | 0 |
|  | Day 1 | 0 | 80 (79.2) | 84 (84.8) | 87 (84.5) |
|  |  | 1 | 17 (16.8) | 11 (11.1) | 9 (8.7) |
|  |  | 3 | 0 | 0 | 1 (1.0) |
|  |  | 4 | 1 (1.0) | 0 | 0 |
|  |  | Missing | 3 (3.0) | 4 (4.0) | 5 (4.9) |
|  | Day 7 | 0 | 91 (90.1) | 91 (91.9) | 93 (90.3) |
|  |  | 1 | 8 (7.9) | 6 (6.1) | 4 (3.9) |
|  |  | 2 | 0 | 0 | 1 (1.0) |
|  |  | Missing | 2 (2.0) | 2 (2.0) | 5 (4.9) |
|  | Day 28 | 0 | 89 (88.1) | 93 (93.9) | 93 (90.3) |
|  |  | 1 | 10 (9.9) | 3 (3.0) | 2 (1.9) |
|  |  | 2 | 0 | 2 (2.0) | 0 |
|  |  | Missing | 2 (2.0) | 1 (1.0) | 8 (7.8) |
| White blood cells, 10_9_/L (decrease) | Baseline | 0 | 89 (88.1) | 91 (91.9) | 93 (90.3) |
|  |  | 1 | 9 (8.9) | 5 (5.1) | 9 98.7) |
|  |  | 2 | 1 (1.0) | 1 (1.0) | 1 (1.0) |
|  |  | Missing | 2 (2.0) | 2 (2.0) | 0 |
|  | Day 1 | 0 | 84 (83.2) | 77 (77.8) | 85 (82.5) |
|  |  | 1 | 12 (11.9) | 17 (17.2) | 11 (10.7) |
|  |  | 2 | 2 (2.0) | 1 (1.0) | 2 (1.9) |
|  |  | Missing | 3 (3.0) | 4 (4.0) | 5 (4.9) |
|  | Day 7 | 0 | 92 (91.1) | 95 (96.0) | 90 (87.4) |
|  |  | 1 | 7 (6.9) | 2 (2.0) | 7 (6.8) |
|  |  | 2 | 0 | 0 | 1 (1.0) |
|  |  | Missing | 2 (2.0) | 2 (2.0) | 5 (4.9) |
|  | Day 28 | 0 | 92 (91.1) | 96 (97.0) | 92 (89.3) |
|  |  | 1 | 7 (6.9) | 2 (2.0) | 3 (2.9) |
|  |  | Missing | 2 (2.0) | 1 (1.0) | 8 (7.8) |
| Absolute neutrophils, 10_9_/L  (decrease) | Baseline | 0 | 60 (59.4) | 64 (64.6) | 65 (63.1) |
|  |  | 1 | 13 (12.9) | 13 (13.1) | 13 (12.6) |
|  |  | 2 | 23 (22.8) | 16 (16.2) | 23 (22.3) |
|  |  | 3 | 3 (3.0) | 4 (4.0) | 2 (1.9) |
|  |  | Missing | 2 (2.0) | 0 | 0 |
|  | Day 1 | 0 | 55 (54.5) | 53 (53.5) | 56 (54.4) |
|  |  | 1 | 19 (18.8) | 20 (20.2) | 13 (12.6) |
|  |  | 2 | 20 (19.8) | 19 (19.2) | 27 (26.2) |
|  |  | 3 | 4 (4.0) | 3 (3.0) | 2 (1.9) |
|  |  | Missing | 3 (3.0) | 4 (4.0) | 5 (4.9) |
|  | Day 7 | 0 | 72 (71.3) | 60 (60.6) | 60 (58.3) |
|  |  | 1 | 12 (11.9) | 18 (18.2) | 15 (14.6) |
|  |  | 2 | 15 (14.9) | 18 (18.2) | 19 (18.4) |
|  |  | 3 | 0 | 1 (1.0) | 4 (3.9) |
|  |  | Missing | 2 (2.0) | 2 (2.0) | 5 (4.9) |
|  | Day 28 | 0 | 52 (51.5) | 51 (51.5) | 53 (51.5) |
|  |  | 1 | 17 (16.8) | 21 (21.2) | 17 (1.5) |
|  |  | 2 | 28 (27.7) | 21 (21.2) | 20 (19.4) |
|  |  | 3 | 2 (2.0) | 5 (5.1) | 5 (4.9) |
|  |  | Missing | 2 (2.0) | 1 (1.0) | 8 (7.8) |
| Absolute lymphocytes, 10_9_/L (decrease) | Baseline | 0 | 98 (97.0) | 96 (97.0) | 102 (99.0) |
|  |  | 1 | 1 (1.0) | 0 | 1 (1.0) |
|  |  | 2 | 0 | 1 (1.0) | 0 |
|  |  | Missing | 2 (2.0) | 0 | 0 |
|  | Day 1 | 0 | 98 (97.0) | 94 (94.9) | 98 (95.1) |
|  |  | 1 | 0 | 1 (1.0) | 0 |
|  |  | Missing | 3 (3.0) | 4 (4.0) | 5 (4.9) |
|  | Day 7 | 0 | 98 (97.0) | 97 (98.0) | 98 (95.1) |
|  |  | 3 | 1 (1.0) | 0 | 0 |
|  |  | Missing | 2 (2.0) | 2 (2.0) | 5 (4.9) |
|  | Day 28 | 0 | 99 (98.0) | 98 (99.0) | 95 (92.2) |
|  |  | Missing | 2 (2.0) | 1 (1.0) | 8 (7.8) |
| Absolute lymphocytes, 10_9_/L (increase) | Baseline | 0 | 94 (93.1) | 93 (93.9) | 94 (91.3) |
|  |  | 2 | 5 (5.0) | 4 (4.0) | 9 (8.7) |
|  |  | Missing | 2 (2.0) | 2 (2.0) | 0 |
|  | Day 1 | 0 | 93 (92.1) | 88 (88.9) | 93 (90.3) |
|  |  | 2 | 5 (5.0) | 7 (7.1) | 5 (4.9) |
|  |  | Missing | 3 (3.0) | 4 (4.0) | 5 (4.9) |
|  | Day 7 | 0 | 84 (83.2) | 82 (82.8) | 86 (83.5) |
|  |  | 2 | 15 (14.9) | 15 (15.2) | 12 (11.7) |
|  |  | Missing | 2 (2.0) | 2 (2.0) | 5 (4.9) |
|  | Day 28 | 0 | 88 (87.1) | 86 (86.9) | 85 (82.5) |
|  |  | 2 | 11 (10.9) | 12 (12.1) | 10 (9.7) |
|  |  | Missing | 2 (2.0) | 1 (1.0) | 8 (7.8) |

Grades are based on the Common Terminology Criteria for Laboratory Abnormalities v5.0.

Supplementary Table 7. Clinical biochemistry.

| Parameter | Time point | Grade | Pyronaridine-artesunate treatment group | | |
| --- | --- | --- | --- | --- | --- |
|  |  |  | 3-day regimen (n=101) | 2-day regimen (n=99) | 1-day regimen (n=103) |
| Total bilirubin, μmol/mL | Baseline | 0 | 97 (96.0) | 91 (91.6) | 102 (99.0) |
|  |  | 1 | 1 (1.0) | 6 (6.1) | 1 (1.0) |
|  |  | 2 | 3 (3.0) | 1 (1.0) | 0 |
|  |  | Missing | 0 | 1 (1.0) | 0 |
|  | Day 1 | 0 | 97 (96.0) | 93 (93.9) | 97 (94.2) |
|  |  | 1 | 3 (3.0) | 4 (4.0) | 1 (1.0) |
|  |  | 2 | 0 | 0 | 0 |
|  |  | Missing | 1 (1.0) | 2 (2.0) | 5 (4.9) |
|  | Day 7 | 0 | 99 (98.0) | 99 (100) | 100 (97.1) |
|  |  | 1 | 2 (2.0) | 0 | 0 |
|  |  | Missing | 0 | 0 | 3 (2.9) |
|  | Day 28 | 0 | 96 (95.0) | 97 (98.0) | 93 (90.3) |
|  |  | 1 | 2 (2.0) | 1 (1.0) | 0 |
|  |  | 2 | 1 (1.0) | 0 | 1 (1.0) |
|  |  | Missing | 2 (2.0) | 1 (1.0) | 9 (8.7) |
| Aspartate aminotransferase, U/L | Baseline | 0 | 100 (99.0) | 91 (91.9) | 101 (98.1) |
|  |  | 1 | 1 (1.0) | 7 (7.1) | 1 (1.0) |
|  |  | Missing | 0 | 1 (1.0) | 1 (1.0) |
|  | Day 1 | 0 | 97 (96.0) | 87 (87.9) | 93 (90.3) |
|  |  | 1 | 3 (3.0) | 6 (6.1) | 4 (3.9) |
|  |  | 2 | 1 (1.0) | 2 (2.0) | 0 |
|  |  | 3 | 0 | 2 (2.0) | 1 (1.0) |
|  |  | Missing | 0 | 2 (2.0) | 5 (4.9) |
|  | Day 7 | 0 | 94 (93.1) | 89 (89.9) | 94 (91.3) |
|  |  | 1 | 7 (6.9) | 9 (9.1) | 6 (5.8) |
|  |  | 2 | 0 | 1 (1.0) | 0 |
|  |  | Missing | 0 | 0 | 3 (2.9) |
|  | Day 28 | 0 | 96 (95.0) | 94 (94.9) | 90 (87.4) |
|  |  | 1 | 3 (3.0) | 4 (4.0) | 5 (4.9) |
|  |  | Missing | 2 (2.0) | 1 (1.0) | 8 (7.8) |
| Alanine aminotransferase, U/L | Baseline | 0 | 101 (100) | 97 (98.0) | 102 (99.0) |
|  |  | 1 | 0 | 1 (1.0) | 1 (1.0) |
|  | Day 1 | 0 | 100 (99.0) | 91 (91.9) | 95 (92.2) |
|  |  | 1 | 1 (1.0) | 5 (5.1) | 2 (1.9) |
|  |  | 2 | 0 | 1 (1.0) | 1 (1.0) |
|  |  | Missing | 0 | 2 (2.0) | 5 (4.9) |
|  | Day 7 | 0 | 97 (96.0) | 91 (91.9) | 99 (96.1) |
|  |  | 1 | 4 (4.0) | 7 (7.1) | 1 (1.0) |
|  |  | 2 | 0 | 1 (1.0) | 0 |
|  |  | Missing | 0 | 0 | 3 (2.9) |
|  | Day 28 | 0 | 99.0 (98.0) | 98 (99.0) | 92 (89.3) |
|  |  | 1 | 0 | 0 | 3 (2.9) |
|  |  | Missing | 2 (2.0) | 1 (1.0) | 8 (7.8) |
| Alkaline phosphatase, U/L | Baseline | 0 | 45 (44.6) | 41 (41.4) | 54 (52.4) |
|  |  | 1 | 51 (50.5) | 51 (51.5) | 46 (44.7) |
|  |  | 2 | 5 (5.0) | 6 (6.1) | 3 (2.9) |
|  |  | Missing | 0 | 1 (1.0) | 0 |
|  | Day 1 | 0 | 49 (48.5) | 47 (47.5) | 48 (46.6) |
|  |  | 1 | 4 (45.5) | 42 (42.4) | 46 (44.7) |
|  |  | 2 | 6 (5.9) | 8 (8.1) | 4 (3.9) |
|  |  | Missing | 0 | 2 (2.0) | 5 (4.9) |
|  | Day 7 | 0 | 49 (49.5) | 49 (49.5) | 53 (51.5) |
|  |  | 1 | 45 (44.6) | 46 (46.5) | 43 (41.7) |
|  |  | 2 | 7 (6.9) | 4 (4.0) | 4 (3.9) |
|  |  | Missing | 0 | 0 | 3 (2.9) |
|  | Day 28 | 0 | 40 (39.6) | 47 (47.5) | 36 (35.0) |
|  |  | 1 | 50 (49.5) | 44 (44.4) | 53 (51.5) |
|  |  | 2 | 9 (8.9) | 7 (7.1) | 6 (5.8) |
|  |  | Missing | 2 (2.0) | 1 (1.0) | 8 (7.8) |

Grades are based on the Common Terminology Criteria for Laboratory Abnormalities v5.0.
